# Reactive Oxygen Species Penetrate Persister Cell Membranes of *Escherichia coli* for Effective Cell Killing

**DOI:** 10.3389/fcimb.2020.00496

**Published:** 2020-09-18

**Authors:** Aki Kawano, Ryota Yamasaki, Tatsuya Sakakura, Yoshiyuki Takatsuji, Tetsuya Haruyama, Yoshie Yoshioka, Wataru Ariyoshi

**Affiliations:** ^1^Division of Infections and Molecular Biology, Department of Health Promotion, Kyushu Dental University, Kitakyushu, Japan; ^2^Division of Functional Interface Engineering, Department of Biological Systems and Engineering, Kyushu Institute of Technology, Kitakyushu, Japan

**Keywords:** reactive oxygen species, persister, *Escherichia coli*, radical vapor reactor, biofilm

## Abstract

Persister cells are difficult to eliminate because they are tolerant to antibiotic stress. In the present study, using artificially induced *Escherichia coli* persister cells, we found that reactive oxygen species (ROS) have greater effects on persister cells than on exponential cells. Thus, we examined which types of ROS could effectively eliminate persister cells and determined the mechanisms underlying the effects of these ROS. Ultraviolet (UV) light irradiation can kill persister cells, and bacterial viability is markedly increased under UV shielding. UV induces the production of ROS, which kill bacteria by moving toward the shielded area. Electron spin resonance-based analysis confirmed that hydroxyl radicals are produced by UV irradiation, although singlet oxygen is not produced. These results clearly revealed that ROS sterilizes persister cells more effectively compared to the sterilization of exponential cells (^**^p < 0.01). These ROS do not injure the bacterial cell wall but rather invade the cell, followed by cell killing. Additionally, the sterilization effect on persister cells was increased by exposure to oxygen plasma during UV irradiation. However, vapor conditions decreased persister cell sterilization by reducing the levels of hydroxyl radicals. We also verified the effect of ROS against bacteria in biofilms that are more resistant than planktonic cells. Although UV alone could not completely sterilize the biofilm bacteria, UV with ROS achieved complete sterilization. Our results demonstrate that persister cells strongly resist the effects of antibiotics and starvation stress but are less able to withstand exposure to ROS. It was shown that ROS does not affect the cell membrane but penetrates it and acts internally to kill persister cells. In particular, it was clarified that the hydroxy radical is an effective sterilizer to kill persister cells.

## Introduction

Commonly used sterilization techniques include autoclaving, ultraviolet (UV) irradiation treatment (Mori et al., 2007), ethylene oxide gas (EOG) exposure, (Shintani, 2017), and radiation sterilization (Goldman and Pruitt, 1998). These techniques are currently used to sterilize medical instruments and food. However, each method has limitations. For example, autoclaving is time-consuming and cannot be used for all materials. In UV irradiation, it is difficult to sterilize areas of an object not exposed to UV light. The EOG method shows residual gas effects after processing, and radiation sterilization is costly and energy-intensive. Thus, a rapid sterilization method that can access all parts of an object, is not toxic, does not produce hazardous waste, is low-cost, and consumes low levels of energy is needed.

Bacteria are resistant to various external stresses such as drugs and starvation by forming “persisters.” Persister bacteria were discovered in 1942 by Hobby et al. (1942). They found that 1% of wild-type *Staphylococcus aureus* survived after penicillin treatment. These cells were named as “persisters” by Bigger (1944). Persister cells are widely present and the phenotype is formed by many types of both Gram-negative and Gram-positive bacteria such as *Escherichia coli* (Balaban et al., 2004), *Pseudomonas aeruginosa* (Fisher et al., 2017), *Enterococcus faecalis* (Abranches et al., 2009; Gaca et al., 2015), *S. aureus* (Corrigan et al., 2016), and *Salmonella enterica* serovar Typhimurium (Helaine et al., 2014; Stapels et al., 2018). Additionally, persister cells account for <0.001% of the cell population in a non-stress environment but can reach as high as 1% in stationary-phase cultures and biofilms (Lewis, 2007, 2008). Persister cells are difficult to completely eliminate because of their high tolerance to stress such as antibiotics treatment (Lewis, 2012; Wood et al., 2019). Slight persister survival allows for cell regrowth and biofilm regeneration (Spoering and Lewis, 2001). Recently, studies on newer medicines to kill persister cells have been reported. For example, it has been reported that two synthetic retinoids (CD437 and CD1530) showed anti-persister activity against a methicillin-resistant *S. aureus* strain (Kim et al., 2018c). Although mitomycin C (Kwan et al., 2015; Cruz-Muniz et al., 2017, 2018) and cisplatin (Chowdhury et al., 2016) also can kill persister cells, these agents have not been approved for clinical treatment (Kim and Wood, 2016). Therefore, persisters remain a problem in the medical and food production fields because they can re-grow in response to external environmental changes and cause serious infectious diseases. To achieve effective sterilization and remove persisters, new sterilization techniques are required.

In our previous study, we developed a radical vapor reactor (RVR) that continuously produces reactive oxygen species (ROS) at high concentrations from water and air (O_2_) which are then exposed to an object. This process is performed at ambient temperature and pressure and exhausts only water and oxygen, as the produced ROS are detoxified by a catalyst after the reaction (Matsuo et al., 2015). Therefore, the RVR has minimal environmental effects. The ROS of the RVR can be used for various applications such as to functionalize the surface of materials (Yamasaki et al., 2017) and for surface cleaning (Yamasaki et al., 2018). ROS are suitable for sterilization because of their strong oxidation activities. The sterilization effects of ROS by RVR have been reported previously (Takatsuji et al., 2017) in a study demonstrating that ROS have stronger sterilization effects than ozone toward exponential cells of *E. coli* (Gram-negative) and *Bacillus subtilis* (Gram-positive). In this present study, we effectively sterilized persister cells using the RVR. Similar to our previous report (Takatsuji et al., 2017), many studies have described sterilization using ROS (Okpara-Hofmann et al., 2005; Murray et al., 2008). However, no studies have evaluated the sterilization of persister cells using ROS externally. To determine whether ROS are effective against persister cells, we prepared rifampicin-induced *E. coli* persister cells as previously described (Kwan et al., 2013; Kim et al., 2018a,b; Yamasaki et al., 2020). The RVR can be used in various modes such as UV mode and O_2_ plasma mode. We aimed to investigate the various RVR modes to evaluate which types of ROS are effective for killing persister cells and the mechanisms underlying these effects.

## Materials and Methods

### Cultivation of *E. coli* and Persister Cell Formation

The *Escherichia coli* K-12 BW25113 strain (Baba et al., 2006) was grown in lysogenic broth (LB; Difco Laboratories, Detroit, MI, USA) (Bertani, 1951) containing 1% (w/v) yeast extract at 37°C. For experiments using exponential cells, the *E. coli* were cultured overnight in a 5-mL LB medium and then inoculated into a 25-mL fresh LB medium at a 1/100 dilution and incubated at 37°C to a turbidity of 0.8 at 600 nm (~2 h). The cells were collected by centrifugation at 3,500 × *g* for 2 min and washed twice with 1× phosphate-buffered saline buffer (PBS) (Dulbecco and Vogt, 1954). The cells were resuspended in 1 mL of 1× PBS. For experiments using persister cells, the cells were grown to a turbidity of 0.8 at 600 nm as described for exponential cells. Rifampicin (FUJIFILM Wako Pure Chemical Corporation, Osaka, Japan) was added at a final concentration of 100 μg/mL, and the culture was incubated at 37°C for 30 min. The antibiotic concentration was selected to be at least 10× the minimum inhibitory concentration, following a previous report (Kwan et al., 2013). The rifampicin-treated culture (5 mL) was harvested by centrifugation at 3,500 × *g* for 10 min and resuspended in 5 mL of LB with ampicillin [final conc. 100 μg/mL, Tokyo Chemical Industry Co., Ltd (Tokyo, Japan)] followed by incubation at 37°C for 3 h to remove non-persister cells. Next, 1 mL of the rifampicin/ampicillin-treated culture was harvested by centrifugation at 3,500 × *g* for 5 min. The cell pellets were washed with 1× PBS at 3,500 × *g* for 2 min and re-suspended in 1 mL of 1× PBS twice. This procedure for developing persister cells was performed as described previously (Kwan et al., 2013; Kim et al., 2018a), and many groups have used this approach (Song and Wood, 2020).

### ROS Treatment by RVR

**Figure 1:**
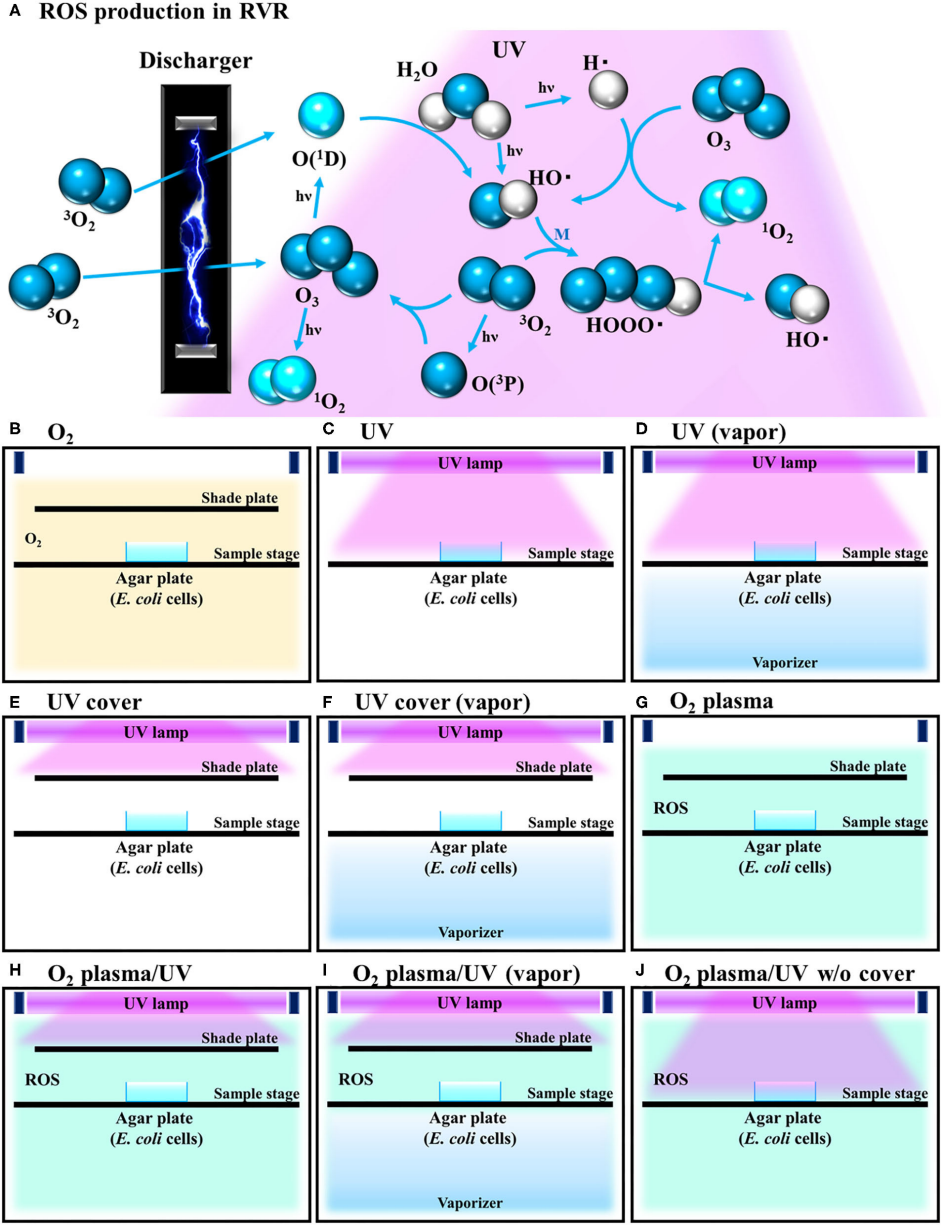
Schematic illustration of reactive oxygen species (ROS) production reaction under various radical vapor reactor (RVR) conditions. **(A)** ROS production reaction pathway. Oxygen is ozonized by discharging. Ozone is degraded to atomic oxygen and singlet oxygen by UV irradiation. Singlet oxygen reacts with H_2_O to produce hydroxyl radicals. HOOO• (hydrogen-trioxide radical) is produced by a reaction between hydroxyl radicals and oxygen, and then cleaved to ^1^O_2_ and HO•. M in the third part of this reaction. **(B)** O_2_ mode as a control. Inserted non-activated O_2_ gas. **(C)** UV mode. Sample on the sample stage was directly exposed only UV. **(D)** UV mode in vapor mode. Internal reactor created high humidity conditions by the vaporizer. **(E)** UV cover mode. UV was irradiated but sample plate was covered with a shade plate to avoid direct irradiation. **(F)** UV cover mode in vapor mode. **(G)** O_2_ plasma mode. Plasmatized ^3^O_2_ molecules by an electric-discharger were introduced into the RVR chamber. Plasmatized (activated) O_2_ plasma produces ROS as shown in **(A)**. **(H)** O_2_ plasma/UV mode (O_2_ plasma mode with UV). **(I)** O_2_ plasma/UV in vapor mode. **(J)** O_2_ plasma/UV no cover mode.

Under all plasma treatment conditions, the temperature in the RVR chamber was set to 40°C. This temperature is suitable for producing singlet oxygen and hydroxy radicals (Matsuo et al., 2015). The following RVR conditions are illustrated in Figures 1B–J. In O_2_ mode, O_2_ was introduced at 4 L/min into the chamber (Figure 1B). In UV mode, the sample on the sample stage was irradiated with UV light (185 and 254 nm) (Figure 1C). In UV (vapor) mode, the sample was irradiated with UV light under humid conditions (vapor) using a stainless-steel dish filled with water as a vaporizer (Figure 1D). In UV cover mode, a shielding plate was used to avoid direct irradiation of the sample (Figure 1E). In UV cover (vapor) mode, UV irradiation was shielded during humidification (Figure 1F). In O_2_ plasma mode, pure oxygen introduced at a constant flow rate into the RVR was plasmatized by an electric discharger (Figure 1G). In O_2_ plasma/UV mode, the sample was exposed to both plasmatized O_2_ and UV simultaneously, but UV was not directly irradiated because of the shielding plate (Figure 1H). In O_2_ plasma/UV (vapor) mode, under humidified conditions, the sample was exposed to both plasmatized O_2_ and UV simultaneously, but UV was not directly irradiated because of the shielding plate (Figure 1I). In O_2_ plasma/UV without (w/o) cover mode, the sample was directly irradiated with UV and plasmatized O_2_ (Figure 1J). Under conditions using UV light, the stainless-steel walls in the RVR chamber were covered with black board to avoid UV light reflection. To avoid nutrient activation of persister cells, M9 agar (no carbon source) (Rodriguez and Tait, 1983) was used for ROS treatment. The cells (8 μL) were plated onto M9 agar, placed on the RVR sample stage, and treated in each RVR mode. After treatment, 30 μL of 10× LB was added to the cell agar plates. These plates were incubated at 37°C overnight and colonies were counted to calculate the survival rate. In the M9 plate with 10× LB, the cells grew, and colonies could be counted as on the LB plate (Supplemental Table 1 and Supplemental Figure 1).

### Electron Spin Resonance Analysis

To detect singlet oxygen and hydroxyl radicals, 2,2,5,5-tetramethly-3-pyrroline-3-carboxamide [TPC, Sigma-Aldrich (St. Louis, MO, USA)] and 5,5-dimethyl-1-pyrroline *N*-oxide [DMPO, LABOTEC (Tokyo, Japan)] were used as spin trap reagents. Each reagent was prepared at 0.5 M in ultrapure water. Next, 1 mL of the prepared reagent solution in the 30-mm culture dish was placed on the center of the sample stage in the RVR. The solution was treated in each RVR mode for 40 or 120 s and then analyzed by electron spin resonance (ESR) spectrometry (JES-FA-100, JEOL, Tokyo, Japan) to detect ROS. The ESR conditions for detecting ROS were as follows: field sweep, 329.1–344.1 mT; field modulation frequency, 100 kHz; field modulation width, 0.05 mT; amplitude, 100; sweep time, 1 min; microwave frequency, 9,450 MHz; microwave power, 4 mW. For calculating the spin-adduct concentration, 0.5 mM to 0.03125 mM 4-hydroxy-2,2,6,6-tetramethyl piperidine-1-oxyl [TEMPOL, Sigma-Aldrich (St. Louis, MO, USA)] was used as a standard sample. The ESR spectrum of Mn^2+^ was used as an internal standard, and the spin concentration was determined using a digital data processor (JEOL, Tokyo, Japan). DMPO-OH and TPC-^1^O_2_ were simulated using the isotropic simulation program. Atmosphere conditions (no RVR treatment) were evaluated as a negative control.

### Scanning Electron Microscopy Observation

The *E. coli* exponential and persister cells were prepared as described above. The cells were suspended in 1 mL 1× PBS and centrifuged twice at 3,500 × *g* for 2 min. The supernatant was discarded, and the pellet was re-suspended in 100 μL 1× PBS. The cell suspensions were plated onto a mixed cellulose esters membrane (Merck Millipore, Billerica, MA, USA) on an M9 agar plate. The cells were treated in each RVR mode for 10 min for complete sterilization. The cells on the membrane were prefixed with 2% glutaraldehyde fixative solution for 1 h. The fixed cell samples were washed twice with 0.1 M phosphate buffer (pH 7.4), and then treated with 50, 70, 90, 95, and 100% acetone for 15 min to dehydrate the samples. For lyophilization, acetone was substituted with *t*-butanol and incubated twice for 30 min at 30°C, and then frozen at −30°C. After lyophilization, platinum deposition was carried out for scanning electron microscopy (SEM) observation (S-4300, HITACHI, Tokyo, Japan).

### Biofilm Removal by RVR

*Escherichia coli* cells were incubated overnight and diluted to a turbidity of 0.05 at 600 nm in LB. The diluted culture was distributed into a 96-well plate (300 μL/well) and incubated for 24 h at 37°C. Planktonic cells were discarded and washed with dH_2_O gently three times. This washing procedure was performed based on a previous biofilm assay report (Zhu et al., 2019). The plate was incubated at 37°C for 20 min to dry. Formed biofilms of *E. coli* were treated in each RVR mode for 10 min. Fresh LB medium was added to each well (300 μL/well) and the plate was incubated for 16 h at 37°C. Wells showing re-growth were counted. Data were analyzed using Excel (Microsoft, Redmond, WA, USA) and expressed as the means ± standard deviations. Differences between multiple groups were assessed by one-way analysis of variance (ANOVA) followed by Tukey's *post-hoc* test. A value of *P* < 0.05 was considered statistically significant.

## Results

### Attenuation of Bactericidal Effect by UV Shading

**Figure 2:**
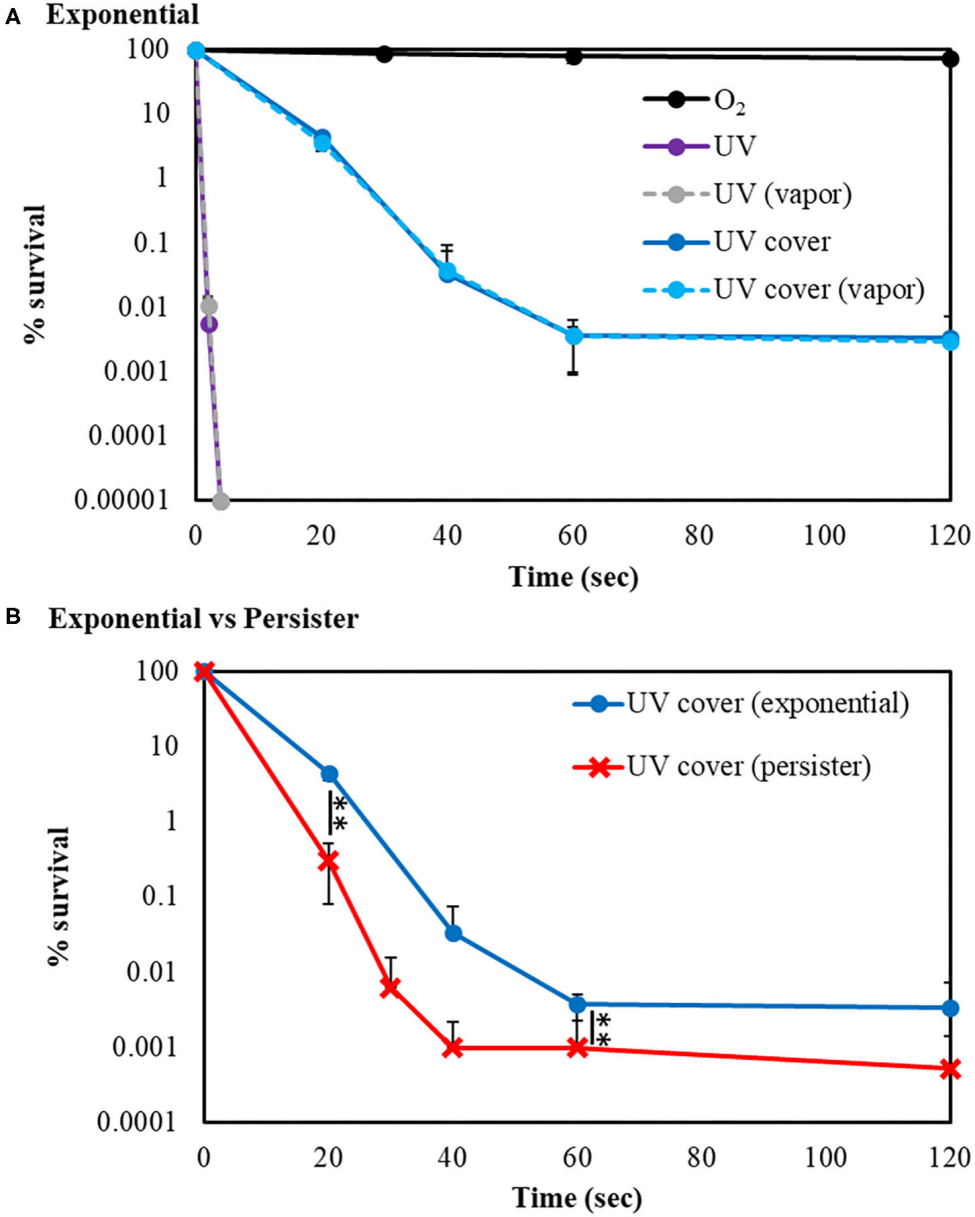
UV sterilization of *E. coli* exponential cells and persister cells with or without cover. **(A)** Survival rate of *E. coli* BW25113 exponential cells after sterilization in UV (purple) or UV cover (blue) modes. Vapor conditions using a vaporizer are indicated as broken lines (gray is UV, and light blue is UV cover). Black plots are O_2_ treatment as a control. **(B)** Comparison of *E. coli* BW25113 exponential cells (blue) and persister cells (red) in UV cover mode treatment. Each exponential or persister cell sample was plated on M9 agar and treated in each RVR mode. Error bars indicate S.D. of at least three experiments. Student's *t*-test was used to compare two groups (**P < 0.01). The values obtained in these experiments are shown in Supplemental Table 2.

**Figure 3:**
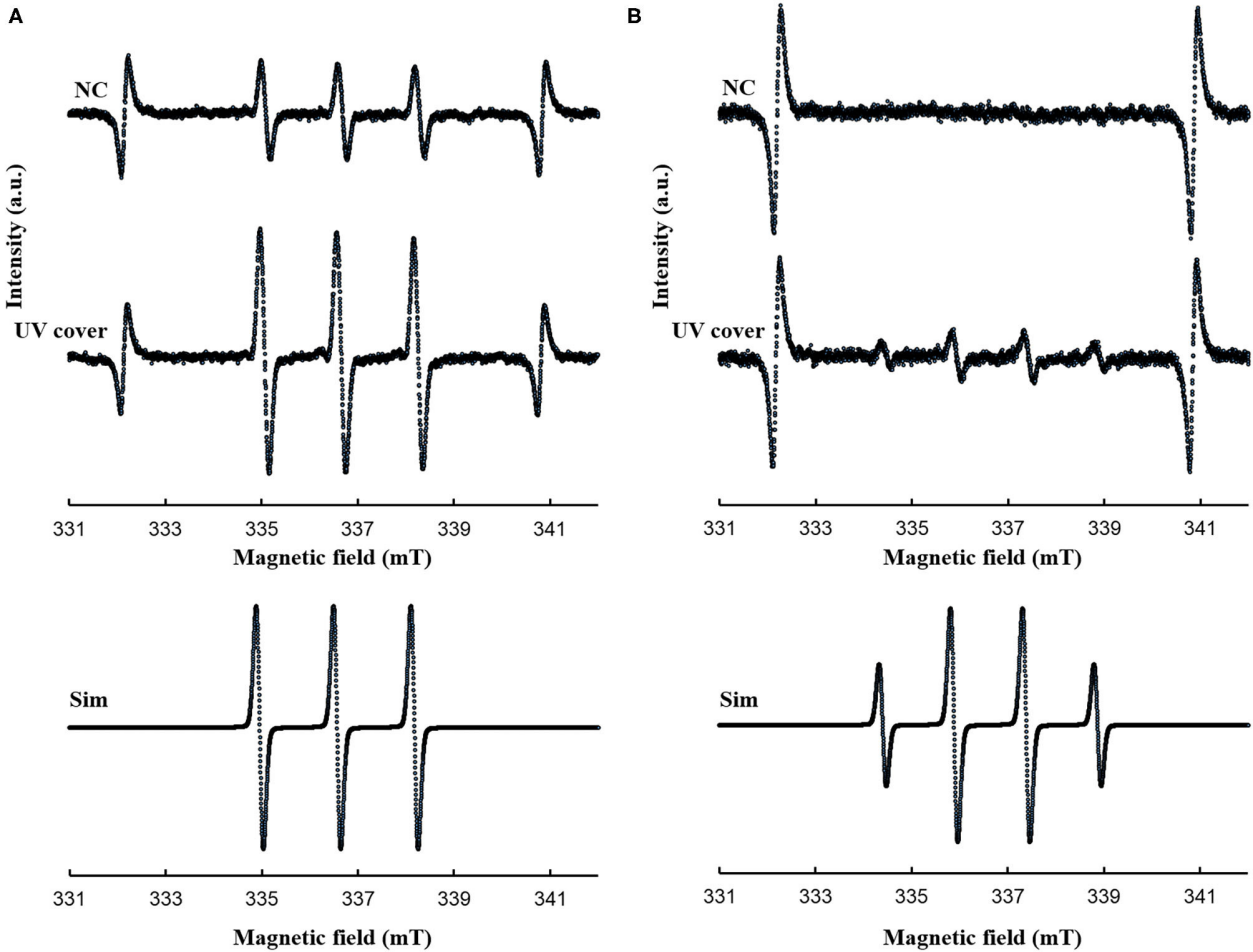
ESR spectra for **(A)** TPC-^1^O_2_ and **(B)** DMPO-OH. A 0.5 mM DMPO or 0.5 mM TPC solution was processed in each RVR condition (NC; negative control and UV cover mode) for 40 sec. Details of the ESR conditions are described in the methods section. Upper spectrum is NC and middle spectrum is UV cover condition. The lower spectra represent the simulated spectra of each adduct. Analyzed values are shown in Table 1. Y-axis indicates intensity (arbitrary unit).

Our developed RVR can produce ROS at high concentrations in a continuous manner as shown in the reaction pathway in Figure 1A. This ROS production reaction pathway was developed based on previous studies (Tasaki et al., 2009; Le Picard et al., 2010; Matsumura et al., 2013; Matsuo et al., 2015; Yamasaki et al., 2017). An RVR can use several modes to combine UV, a discharger, and vaporizer (Figures 1B–J). First, the *E. coli* bactericidal effects in UV mode (Figure 1C), UV mode with vapor (Figure 1D), UV cover mode (Figure 1E), or UV cover mode with vapor (Figure 1F) were investigated. Direct irradiation with UV immediately killed the bacterial cells in a few seconds (Figure 2A). However, when direct UV irradiation was blocked by the cover, the bactericidal effect was greatly decreased (Figure 2A); however, *E. coli* died over an area of a few inches despite the shielding of UV light. We predicted that ROS were produced by UV and moved toward the sample. In each UV or UV cover mode, no differences in sterilizing effects were observed in the presence and absence of vapor (high or low humidity) (Figure 2A). Next, *E. coli* persister cells or exponential cells induced by rifampicin (Kwan et al., 2013) were treated in UV cover mode. As shown for the exponential cells in Figure 2A, direct irradiation with UV immediately killed all persister cells (Supplemental Figure 2). Notably, UV cover mode showed an ~10-fold higher bactericidal effect on persister cells than on exponential cells for 120 s treatment (Figure 2B). After 40 s of treatment, a 100-fold higher bactericidal effect on persister cells than on exponential cells was observed. Therefore, ROS by UV irradiation efficiently killed persister cells rather than exponential cells. This result was statistically significant (^**^p < 0.01). The values obtained in these experiments are shown in Supplemental Table 2. To identify the ROS produced by UV, two primary types of ROS (singlet oxygen and hydroxyl radical) that can be detected by ESR were analyzed. Each spin trap reagent was placed on the sample stage in RVR for 40 s as the negative control (NC). To detect singlet oxygen, TPC is a suitable reagent (Nakamura et al., 2011). Singlet oxygen by TPC was weakly detected and UV cover mode detected specific signal of TPC-^1^O_2_ more than the NC (Figure 3A). Hydroxyl radical by DMPO was detected in less quantity and UV cover mode showed a specific signal of DMPO-OH (1:2:2:1 quartet pattern intensity) (Nakamura et al., 2010) (Figure 3B). Both TPC-^1^O_2_ and DMPO-OH were simulated and are shown in Figure 3 (lower spectra). For each simulation, the following hyperfine coupling constants were used: a_N_ = a_H_ = 1.61 mT and g = 2.0061 (Matsumura et al., 2013) for TPC-^1^O_2_, and a_N_ = 15 mT, a_H_ = 14.7 mT, and g = 2.0057 for DMPO-OH (Bosnjakovic and Schlick, 2006). Each ROS concentration was obtained by analyzing ESR spectra and is shown in Table 1. As shown in Table 1, 4.4-fold higher levels of hydroxyl radicals were detected in UV cover mode than in the NC. Singlet oxygen levels were also 2.2-fold higher than in the NC (Table 1).

**Table 1:** Reactive oxygen species (ROS) analysis using electron spin resonance (ESR) after treatment in UV cover mode.

**Sample**	**Concentration**		**Fold-change**	
	**^1^O_2_ (μM)**	**HO· (nM)**	**^1^O_2_**	**HO·**
NC	7 ± 3	90 ± 40	1	1
UV cover	16 ± 1	400 ± 100	2.2	4.4

*The solution of the spin trap reagent (0.5 M TPC or 0.5 M DMPO) was treated in UV cover mode for 40 s. The solution sample was analyzed by ESR to detect ROS. Singlet oxygen (^1^O_2_) is mainly trapped by TPC and hydroxyl radical (HO·) is mainly trapped by DMPO. Fold-changes are based on atmospheric conditions as a negative control (NC). These data were obtained from at least three different experiments. ESR spectra are presented in Figure 3*.

### Effect of ROS on Bacterial Cell Wall

**Figure 4:**
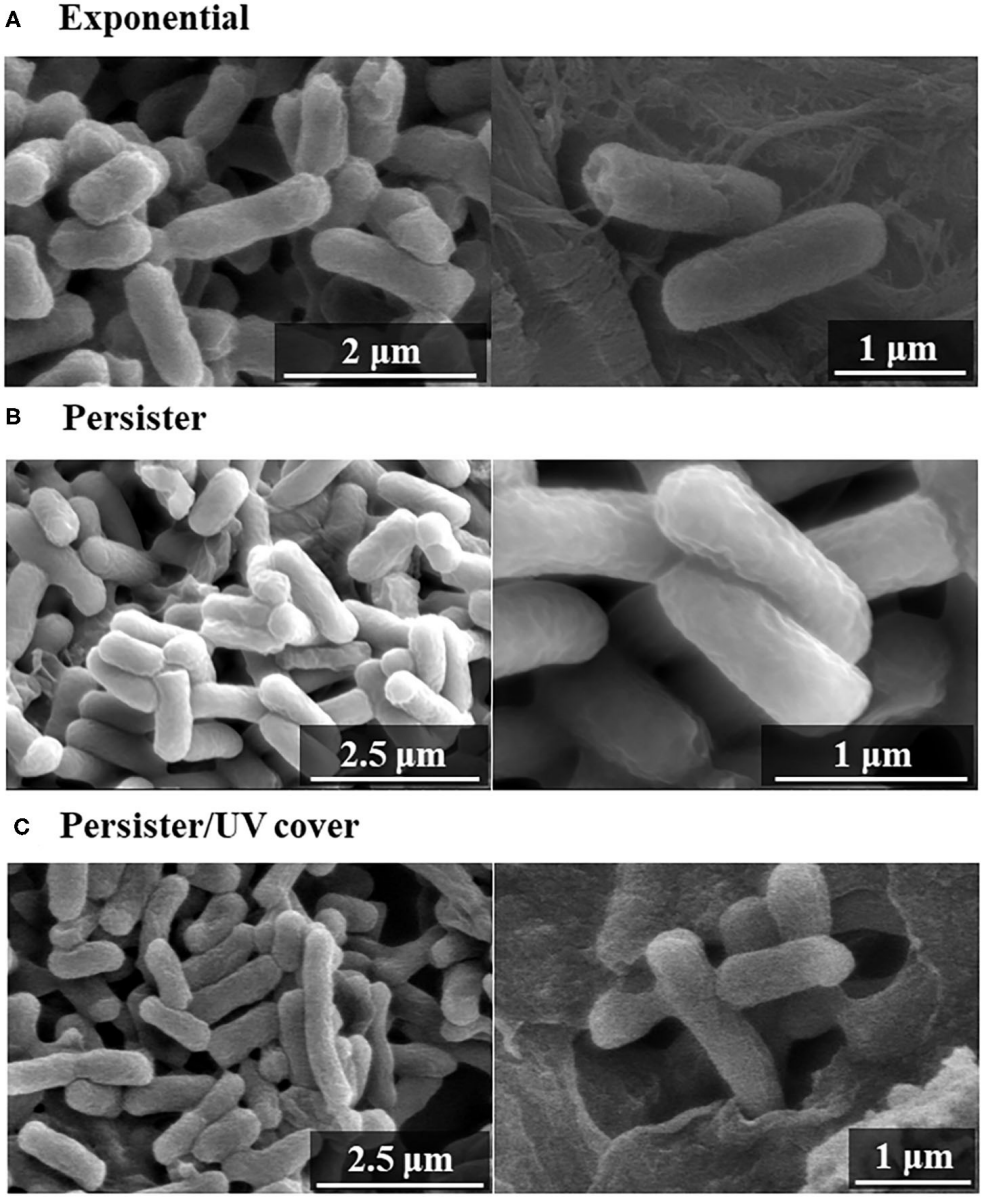
Scanning electron microscopy (SEM) images after UV irradiation. Bacterial cell wall observation of **(A)** exponential cells, **(B)** persister cells, and **(C)** persister cells treated with UV with cover for 10 min (UV cover) by SEM.

To determine how bactericidal effects by UV cover treatment affect the cell wall, the *E. coli* cell surface was observed by SEM after treatment. Both *E. coli* exponential cells and persister cells showed nearly the same regular cell wall surface (Figures 4A,B). The *E. coli* persister cells after treatment in UV cover mode are shown in Figure 4C. To determine the efficiency of the effects of ROS, persister cells were treated in UV cover mode for 10 min [this time is excessive, as 40 s of treatment has a 99.99% sterilization effect (Figure 2B)]. Although lethal treatment was applied, no noticeable injury was found (Figure 4C). This result clearly indicates that ROS kill bacteria without injuring the bacterial cell wall.

### Sterilization Effect of Other RVR Modes

**Figure 5:**
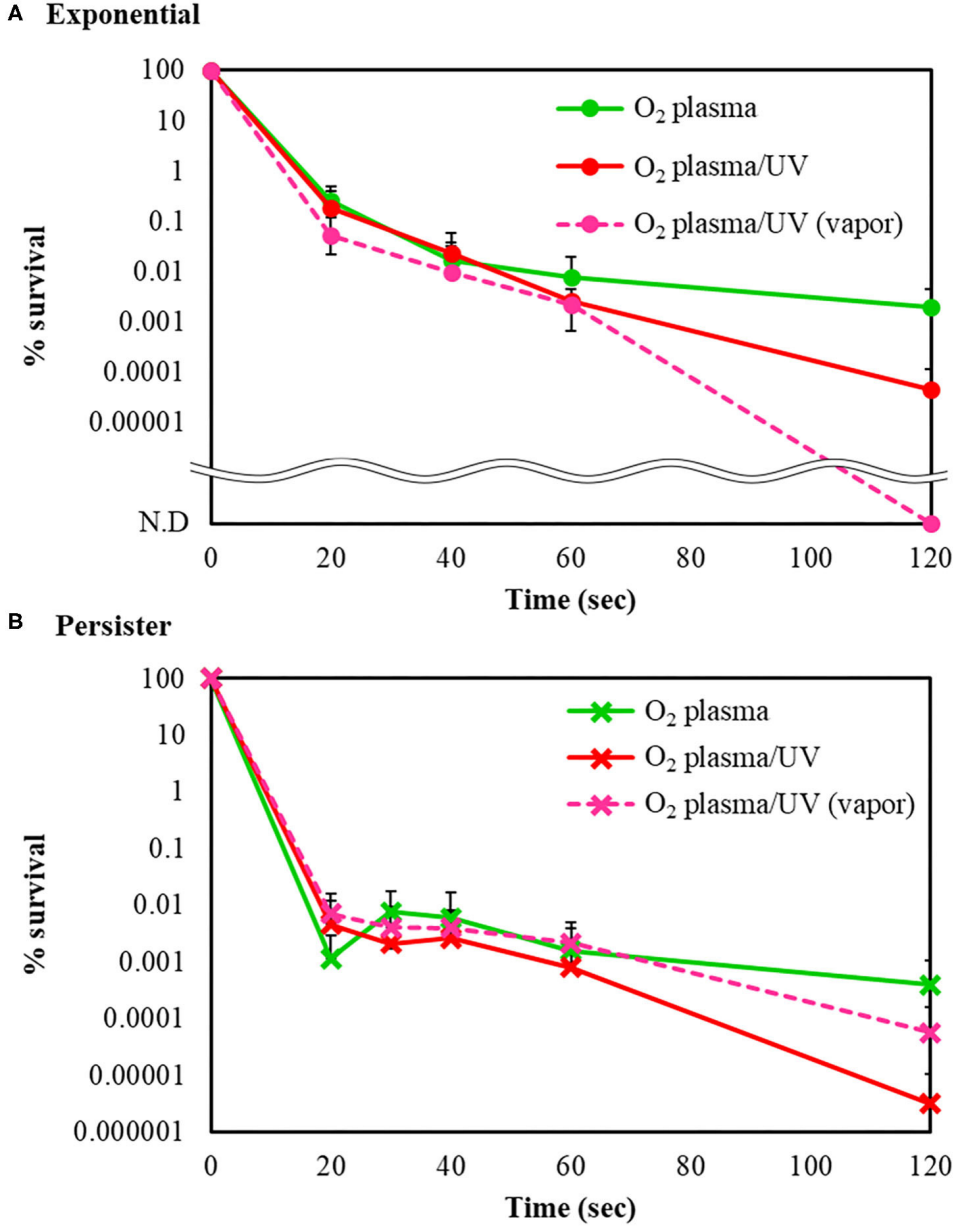
Bactericidal effects of other radical vapor reactor (RVR) conditions. Survival rate of **(A)** *E. coli* exponential cells and **(B)** *E. coli* persister cells after sterilization with O_2_ plasma (green), O_2_ plasma/UV (red), and O_2_ plasma/UV with vapor (pink, broken line) modes. Each exponential or persister cell sample was plated on M9 agar and treated in each RVR mode. Non-detectable bacterial numbers are indicated as N.D. Error bars indicate S.D. of at least three experiments. The values obtained in these experiments are shown in Supplemental Table 3.

**Figure 6:**
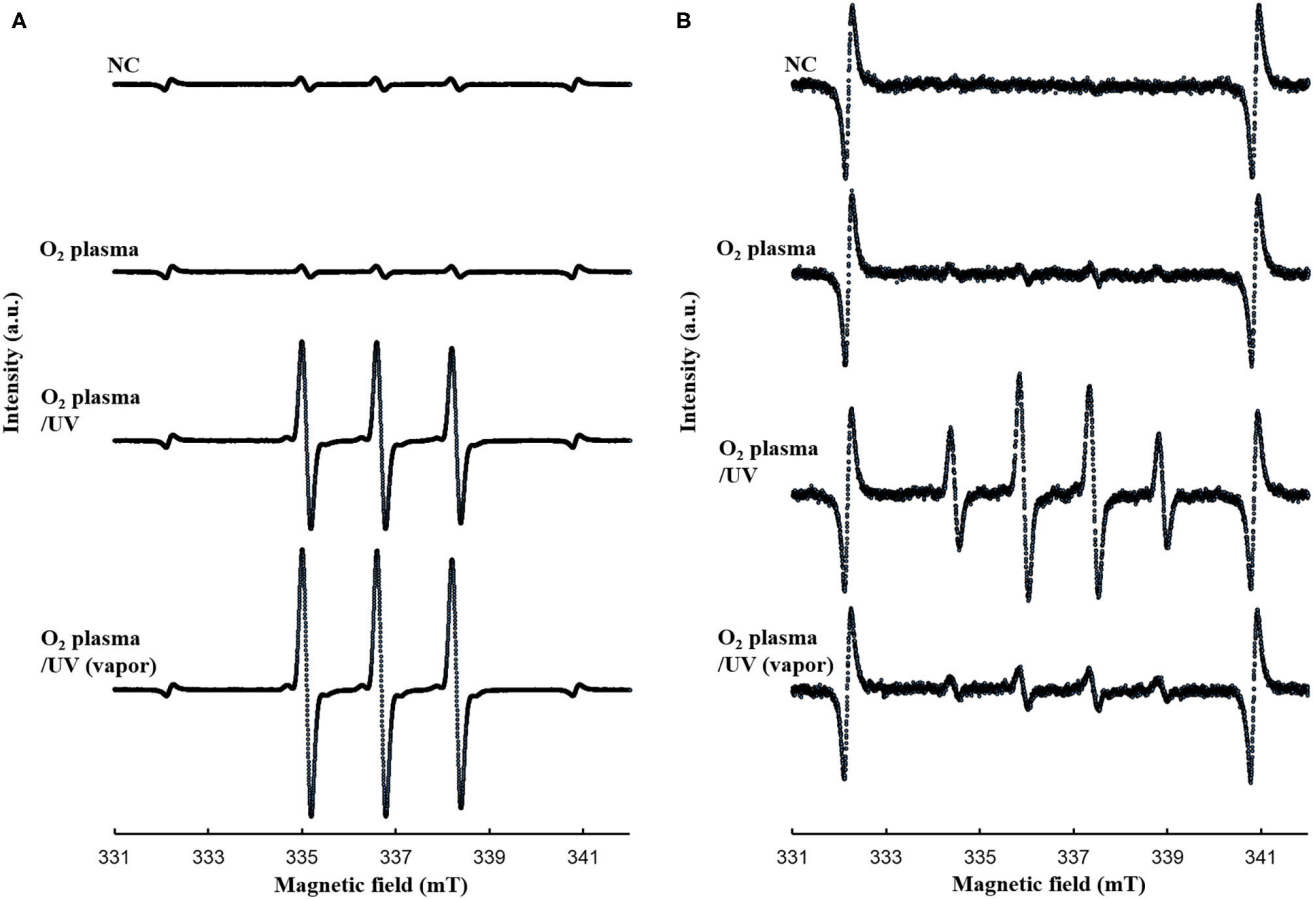
ESR spectra for **(A)** TPC-^1^O_2_ and **(B)** DMPO-OH. A 0.5 mM DMPO or 0.5 mM TPC solution was processed in each RVR condition [NC, O_2_ plasma, O_2_ plasma/UV, and O_2_ plasma/UV (vapor)] for 120 sec. Details of the ESR conditions are provided in the methods section. NC, O_2_ plasma, O_2_ plasma/UV, and O_2_ plasma/UV (vapor) condition are indicated. Analyzed values are shown in Table 2. Y-axis indicates intensity (arbitrary unit).

The bactericidal effects of other RVR modes on *E. coli* exponential and persister cells were investigated. The RVR can produce O_2_ plasma by discharging O_2_ gas. O_2_ plasma mode alone, O_2_ plasma/UV mode (combining O_2_ plasma and UV irradiation), and O_2_ plasma/UV with vapor in vaporizer mode were evaluated (Figures 1B,G–I). For exponential cells, O_2_ plasma/UV with vapor showed good bactericidal effects (Figure 5A). All cells were sterilized at O_2_ plasma/UV with vapor for 120 s. In contrast, O_2_ plasma/UV without vapor was more effective than O_2_ plasma/UV with vapor against persister cells (Figure 5B). The values obtained in these experiments are shown in Supplemental Table 3. Both singlet oxygen and hydroxyl radicals produced in each mode were quantified by ESR. In O_2_ plasma mode, the spectral intensity of ESR was almost the same as that for NC (Figure 6A). In contrast, O_2_ plasma/UV and O_2_ plasma/UV with vapor modes showed higher signal than O_2_ plasma mode (Figure 6A). Although the NC did not detect the hydroxyl radical, the O_2_ plasma mode detected and showed a specific signal (Figure 6B). The O_2_ plasma/UV mode showed a highly specific signal. However, the O_2_ plasma/UV with vapor mode decreased the DMPO-OH signal (Figure 6B). Each ROS concentration was obtained by analyzing ESR spectra and is shown in Table 2. In a comparison of O_2_ plasma/UV and O_2_ plasma/UV with vapor, the levels of singlet oxygen in both modes were very high (31–and 37-fold) but not significantly different (Table 2). However, O_2_ plasma/UV produced 3-fold higher hydroxy levels compared to O_2_ plasma/UV with vapor (Table 2). In contrast, in O_2_ plasma mode, hydroxyl radicals were detected; however, singlet oxygen was not detected (Table 2).

**Table 2:** Reactive oxygen species (ROS) analysis using electron spin resonance (ESR) in various radical vapor reactive modes.

**Sample**	**Concentration**		**Fold-change**	
	**^1^O_2_ (μM)**	**HO· (nM)**	**^1^O_2_**	**HO·**
NC	6 ± 2	100 ± 50	1	1
O_2_ plasma	7 ± 3	200 ± 70	1	2
O_2_ plasma/UV	200 ± 40	900 ± 200	31.2	9
O_2_ plasma/UV (vapor)	230 ± 8	310 ± 40	36.9	3.1

*The solution of the spin trap reagent (0.5 M TPC or 0.5 M DMPO) was treated in O_2_ plasma, O_2_ plasma/UV, and O_2_ plasma/UV with vapor mode for 120 s. Fold-changes are based on atmospheric conditions as a negative control (NC). These data were obtained from at least three experiments. ESR spectra are presented in Figure 6*.

### *Escherichia coli* Biofilm Removal by RVR

**Figure 7:**
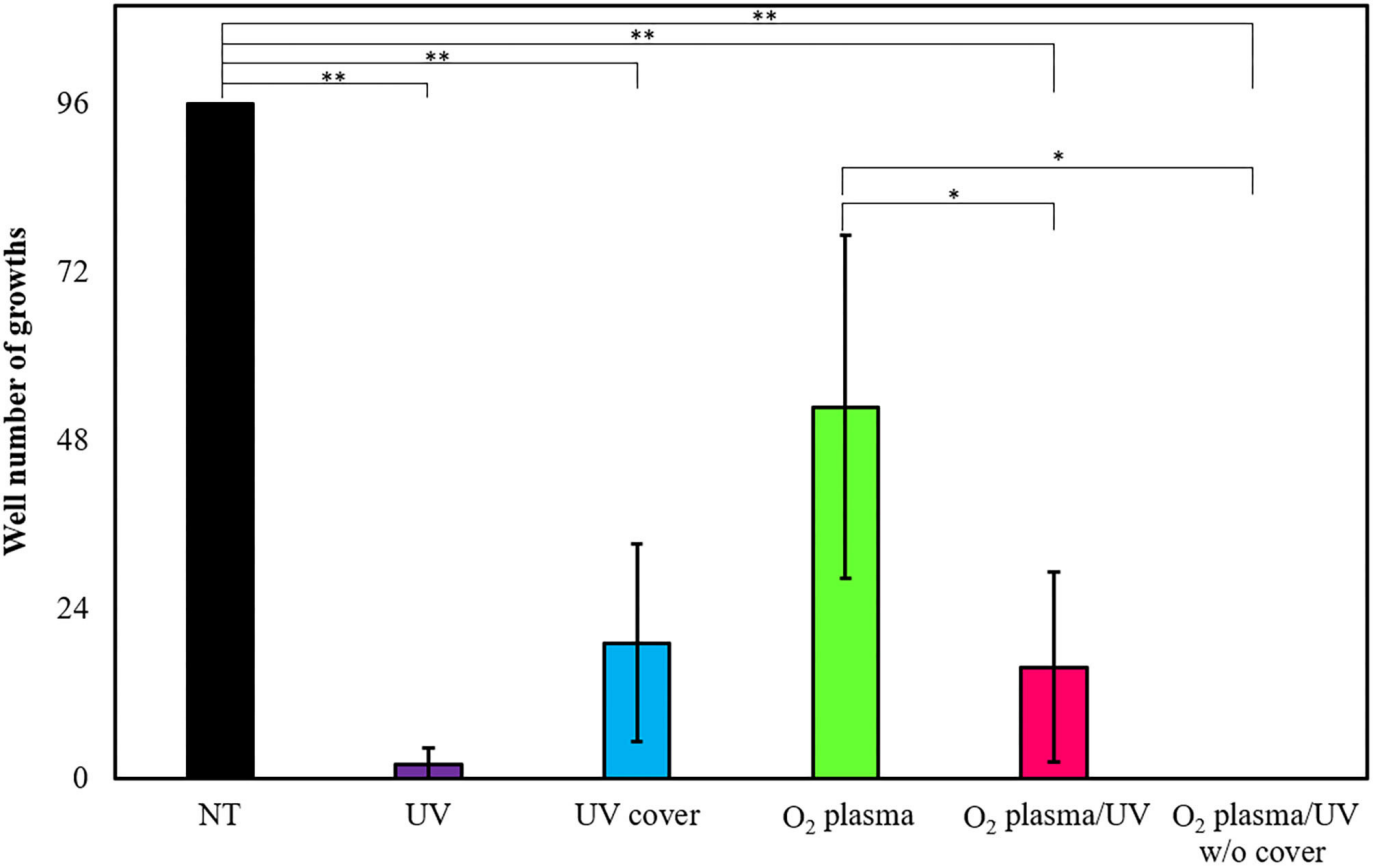
Biofilm extinction by radical vapor reactor (RVR) treatment. Number of wells growing cells after treatment in UV (purple), UV cover (blue), O_2_ plasma (green), O_2_ plasma/UV (pink), and O_2_ plasma/UV without cover mode. Formed *E. coli* biofilms on the 96-well plate were treated in each RVR mode for 10 min, and then LB medium was added to all wells and incubated at 37°C for 16 h. Wells which re-grew cells were counted. Non-treatment (NT) as a control is indicated at a black bar. Error bars indicate S.D. of at least three experiments. All data were analyzed using Tukey's *post-hoc* test after one-way analysis of variance. Significant difference of two individual groups comparison connects with a line and indicates at asterisks (*P < 0.05, **P < 0.01). The values obtained in these experiments are shown in Supplemental Table 4.

*Escherichia coli* forms a biofilm during growth. Biofilm cells are generally considered as more resistant to treatment than planktonic cells (Bridier et al., 2011). Therefore, the effects of various RVR modes on *E. coli*-derived biofilms were examined and tested to ascertain whether the biofilms could be completely sterilized. *E. coli* biofilms formed on the 96-well plate were each treated with UV cover, UV, O_2_ plasma, O_2_ plasma/UV, and O_2_ plasma/UV without cover. Non-treatment (NT) as a control (wash alone) grew in all 96 wells (Figure 7). Hence, there was no effect of washing against biofilm bacteria. Interestingly, UV irradiation for 10 min of treatment could not completely remove the biofilm (2% survived), whereas O_2_ plasma/UV without a cover killed all biofilm cells (Figure 7). UV cover and O_2_ plasma/UV modes showed nearly the same results (19 and 16% survived, respectively). The O_2_ plasma mode did not effectively remove the biofilm (53% survived). The values obtained in these experiments are shown in Supplemental Table 4.

## Discussion

Persister cells are highly tolerant to stress compared to cells in the exponential state. Therefore, sterilization of persister cells is difficult. In this study, we found that ROS (particularly hydroxyl radicals) effectively sterilize *E. coli* persister cells compared to exponential cells. Thus, this sterilization method is suitable depending on the bacterial phenotype. Thus, RVR has fewer disadvantages than EOG and radiation sterilization and shows highly effective sterilization. These results may be useful for developing effective sterilization methods for use in the public health and clinical medicine fields.

Previously, Vermeulen et al. (2008) reported the growth inhibitory effect of UV light in *E. coli*. When irradiated with various wavelengths of radiation, a wavelength of 265 nm was shown to be the most effective for killing of *E. coli*. Our study demonstrated that UV was effective for killing both *E. coli* exponential cells and persister cells in <5 s (Figure 2A and Supplemental Figure 2). As shown in Figure 2A, the germicidal effect was reduced when UV irradiation was blocked with a shielding plate. Therefore, this result suggests that bacteria cell survival to avoid UV light is a limitation of UV treatment. However, despite the complete inhibition of UV irradiation toward the cell sample, the levels of surviving *E. coli* were gradually decreased. ROS are produced by UV irradiation to oxygen and water in the atmosphere (de Jager et al., 2017; Georgiou et al., 2017). In the ESR results, hydroxyl radicals were detected at high concentrations than in the NC. Because ROS are produced by UV, bacteria are not enough but killed. *E. coli* persister cells were killed at ~100-fold higher levels than exponential cells by 40-s treatment in UV cover mode. Singlet oxygen and hydroxyl radicals were present at higher levels than in the NC in UV cover mode. Therefore, ROS, particularly hydroxyl radicals, have greater effects on persister cells than on exponential cells. To determine if ROS injures the bacterial cell membrane and kills the cells directly or penetrates the intracellular environment to kill the cells, *E. coli* exponential, persister, and persister cells after treatment in UV cover mode were observed by SEM (Figure 4). No large differences in cell shape were observed. ROS did not react with the bacterial cell wall but rather reacted intracellularly to exert cell-killing effects. Oxidizing bactericidal agents are widely used to kill pathogenic bacteria, and many studies of the characteristics and mechanisms of their action have been reported (Linley et al., 2012; Vatansever et al., 2013). In general, intracellularly produced ROS exert bactericidal action by causing DNA damage, protein denaturation, and lipid peroxide production, which negatively impact cell survival (Cabiscol et al., 2000; Dwyer et al., 2007; Hong et al., 2017; Van Acker and Coenye, 2017). Therefore, ROS produced during RVR killed the bacteria.

ROS effectively killed persister cells. The RVR can produce high levels of ROS to plasmatize O_2_ gas. This O_2_ plasma combined UV cover mode also showed sterilization effects. High humidity conditions (with vapor) were also evaluated, as ROS production may be altered by H_2_O. Interestingly, the opposite results were obtained for exponential cells and persister cells (Figure 5). For exponential cells, O_2_ plasma/UV with vapor showed greater sterilization effects than without vapor (Figure 5A). In contrast, O_2_ plasma/UV with vapor showed lower sterilization effects than without vapor for persister cells (Figure 5B). Comparison of the concentration of ROS under each condition revealed that singlet oxygen was produced at the same high level in both O_2_ plasma/UV without vapor mode and O_2_ plasma/UV with vapor mode, and the hydroxyl radical level was 3-fold higher in O_2_ plasma/UV without vapor mode than in O_2_ plasma/UV with vapor mode (Table 2). Therefore, high concentrations of hydroxyl radical showed strong sterilization effects toward persister cells, and high concentrations of singlet oxygen and low concentrations of hydroxyl radical showed strong sterilization effects toward exponential cells. A previous study also indicated that the vapor condition had higher sterilization effects on cells in the exponential state than the condition without vapor (Takatsuji et al., 2017). It has been reported that viable *E. coli* persister cells can stop the production of hydroxyl radicals (Kim et al., 2011). Thus, hydroxyl radicals strongly affect the survival of persister cells. In the absence of UV (O_2_ plasma mode), sterilization effects were weak toward both cell types. Because O_2_ plasma exposure alone did not result in high production of ROS, this method is less effective than O_2_ plasma/UV mode. In addition, O_2_ plasma/UV mode was continuously performed to completely sterilize the persister cells. All persister cells were killed after 360 s of treatment (Supplemental Figure 3).

We demonstrated the RVR sterilization effect dispersed not only *E. coli* but also the biofilm formed by *E. coli* (Figure 7). Direct UV irradiation immediately killed (<5 s) the cells on an agar plate. However, UV could not completely kill biofilm cells even after irradiation for 10 min. UV sterilization effects weaken as the distance between the UV lamp and sample increases (Bank et al., 1990). Additionally, areas not reached by UV are difficult to irradiate, and small samples are difficult to sterilize. Thus, cells deeply positioned in the biofilm on the 96-well plate may have not been sterilized and then re-grew. The poor sterilization effect of O_2_ plasma mode (green) is due to the low production of both singlet oxygen and hydroxyl radicals (Table 2). The UV cover mode (blue) and O_2_ plasma/UV (pink) mode showed better sterilization effects than O_2_ plasma, as the ROS concentration was higher; however, the sterilization effects were low. There is no statistical significance between UV cover mode (blue) and O_2_ plasma/UV mode (pink), but O_2_ plasma/UV showed higher effect than UV cover mode. This result also related in ROS amount. Importantly, O_2_ plasma/UV mode without a cover showed complete sterilization effects. This mode directly combined UV irradiation and O_2_ plasma exposure. The ROS produced by O_2_ plasma also reached deep parts of the sample, enabling complete sterilization. These results suggested that the biofilm, that is, both exponential cells and the persister, can be sterilized in a short time by using RVR.

In summary, this study revealed that hydroxyl radicals have strong bactericidal effects against persister cells, which cause intractable infection diseases. Combining UV and O_2_ plasma resulted in strong sterilization effects against biofilm. ROS, particularly hydroxyl radicals, effectively remove persister cells and biofilm remaining even after chemical treatment. The time required for sterilization by the RVR is much shorter than that for autoclaving sterilization. Furthermore, no hazardous waste is produced, and RVR sterilization is not costly, as it only requires oxygen gas. Thus, ROS produced by RVR may be more effective for sterilizing persisters compared to conventional sterilization methods and may be useful for performing sterilization operations in the medical field. We will use the RVR for sterilization of dental instruments. Some dental instruments cannot be autoclaved and, thus, require sterilization. For these instruments, EOG or UV sterilization methods have been used. However, the EOG method shows residual gas effects after processing and the UV method cannot completely kill bacteria. In contrast, RVR can use a wide variety of instruments and achieve complete sterilization in a short time (a few seconds). Thus, RVR may become an alternative general sterilization method.

## Data Availability Statement

All datasets generated for this study are included in the article/Supplementary Material.

## Author Contributions

AK, RY, TS, and YT performed the experiments. AK wrote the initial draft of the manuscript. RY assisted in the preparation of the manuscript. AK, RY, TS, YT, TH, YY, and WA designed the experiments. All authors have contributed to data collection, interpretation, critically reviewed the manuscript, read, and approved the manuscript.

## Conflict of Interest

The authors declare that the research was conducted in the absence of any commercial or financial relationships that could be construed as a potential conflict of interest.

## Supplementary Material

Supplemental Figure 1 Cell growth comparison on the LB plate and LB with M9 plate. *Escherichia coli* persister cells were diluted at 10^5^ and plated (8 μL) onto the LB plate or M9 plate. Next, 30 μL of 10× LB was added to the M9 cell agar plates. These plates were incubated at 37°C for 1–2 days.

Supplemental Figure 2 UV effect to the persister cells. Comparison of the bactericidal effect by UV directly irradiation (UV mode) to *E. coli* exponential cells (blue) and persister cells (red).

Supplemental Figure 3 Complete sterilization of persister cells using O_2_ plasma/UV mode. Longer time treatment (360 s) in O_2_ plasma/UV mode of persister cells (Figure 5B).

Supplemental Table 1 Cell growth comparison on the LB plate and M9 with LB plate. *Escherichia coli* persister cells were diluted at 10^5^ and plated (8 μL) onto the LB plate or M9 plate. Next, 30 μL of 10x LB was added to the M9 cell agar plates. These plates were incubated at 37°C for 1–2 days.

Supplemental Table 2 Number of survivingcells after RVR treatment. Colony forming units of *E. coli* after treatment in UV or UV cover mode were counted (CFU/mL). The graph of these data is shown in Figure 2.

Supplemental Table 3 Number of surviving cells after RVR treatment. Colony forming units of *E. coli* after treatment in O_2_ plasma or O_2_ plasma/UV mode were counted (CFU/mL). The graph of these data is shown in Figure 4.

Supplemental Table 4 *Escherichia coli* biofilm removal by RVR. The well number of growths after treatment in UV, UV cover, O_2_ plasma, O_2_ plasma/UV, and O_2_ plasma/UV without cover mode. Non-treatment (NT) is indicated as a control. The graph of these data is shown in Figure 5.
