# Supplementary material for: Reactive Oxygen Species Penetrate Persister Cell Membranes of Escherichia coli for Effective Cell Killing
Source: Front Cell Infect Microbiol. 2020 Sep 18;10:496. doi: 10.3389/fcimb.2020.00496 (PMC7530241; doi:10.3389/fcimb.2020.00496)
Supplement: Supplemental Figure 1 — Cell growth comparison on the LB plate and LB with M9 plate. Escherichia coli persister cells were diluted at 105 and plated (8 μL) onto the LB plate or M9 plate. Next, 30 μL of 10× LB was added to the M9 cell agar plates. These plates were incubated at 37°C for 1–2 days. [file Data_Sheet_1.docx]

**SUPORTING INFORMATION**

**Reactive oxygen species penetrate persister cell membranes for effective cell killing**

**Aki Kawano**^1^**, Ryota Yamasaki**^1*^, **Tatsuya Sakakura**^2^**, Yoshiyuki Takatsuji**^2^**, Tetsuya Haruyama**^2^**, Yoshie Yoshioka**^1^**, Wataru Ariyoshi**^1^

^1^Division of Infections and Molecular Biology, Department of Health Promotion, Kyushu Dental University, Kitakyushu, Fukuoka, Japan

^2^Division of Functional Interface Engineering, Department of Biological Systems and Engineering, Kyushu Institute of Technology, Kitakyushu, Fukuoka, Japan

***Correspondence:**

Dr. Ryota Yamasaki

E-mail r18yamasaki@fa.kyu-dent.ac.jp

**Keywords: reactive oxygen species, persister, *Escherichia coli*, radical vapor reactor, biofilm**

**Supplemental Table 1. Cell growth comparison on the LB plate and M9 with LB plate.** *Escherichia coli* persister cells were diluted at 10^5^ and plated (8 µL) onto the LB plate or M9 plate. Next, 30 µL of 10x LB was added to the M9 cell agar plates. These plates were incubated at 37°C for 1–2 days.

| **Plates** | **Microorganism counts (log_10_(CFU/mL))** | |
| --- | --- | --- |
|  | **overnight** | **2days** |
| **LB** | 8.38 ± 0.08 | 8.38 ± 0.08 |
| **M9** | 8.31 ± 0.28 | 8.31 ± 0.28 |

**Supplemental Table 2. Number of surviving cells after RVR treatment.** Colony forming units of *E. coli* after treatment in UV or UV cover mode were counted (CFU/mL). The graph of these data is shown in **Fig. 2**.

| **Time (sec)** | **0** | **2** | | **4** | |  |  |  |  |
| --- | --- | --- | --- | --- | --- | --- | --- | --- | --- |
| **UV** | 4.3 × 10^8^ ± 2.0 × 10^8^ | 3.5 × 10^4^ ± 5.1 × 10^4^ | | 0 | |  |  |  |  |
| **UV (vapor)** | 4.3 × 10^8^ ± 2.0 × 10^8^ | 6.6 × 10^4^ ± 2.8 × 10^4^ | | 0 | |  |  |  |  |
| **Time (sec)** | **0** | | **20** | | **30** | | **40** | **60** | **120** |
| **O_2_** | 7.1 × 10^8^ ± 3.4 × 10^7^ | | - | | 6.1 × 10^8^ ± 4.0 × 10^7^ | | - | 5.7 × 10^8^ ± 1.5 × 10^8^ | 5.3 × 10^8^ ± 5.7 × 10^7^ |
| **UV cover** | 7.7 × 10^8^ ± 1.2 × 10^8^ | | 3.6 × 10^7^ ± 4.0 × 10^6^ | | - | | 2.3 × 10^5^ ± 2.7 × 10^5^ | 3.0 × 10^4^ ± 1.1 × 10^4^ | 2.5 × 10^4^ ± 2.8 × 10^4^ |
| **UV cover (vapor)** | 7.7 × 10^8^ ± 1.2 × 10^8^ | | 2.9 × 10^7^ ± 7.4 × 10^6^ | | - | | 2.8 × 10^5^ ± 3.9 × 10^5^ | 2.7 × 10^4^ ± 1.9 × 10^4^ | 2.2 × 10^4^ ± 3.1 × 10^4^ |
| **UV cover [persister]** | 4.2 × 10^8^ ± 1.2 × 10^8^ | | 1.8 × 10^6^ ± 1.5 × 10^6^ | | 3.3 × 10^4^ ± 4.7 × 10^4^ | | 5.0 × 10^3^ ± 5.8 × 10^3^ | 4.4 × 10^3^ ± 5.0 × 10^3^ | 2.5 × 10^3^ ± 3.9 × 10^3^ |

**Supplemental Table 3. Number of surviving cells after RVR treatment.** Colony forming units of *E. coli* after treatment in O_2_ plasma or O_2_ plasma/UV mode were counted (CFU/mL). The graph of these data is shown in **Fig. 4**.

| **Exponential cells** |  |  |  |  |  |  |
| --- | --- | --- | --- | --- | --- | --- |
| **Time (sec)** | **0** | **20** |  | **40** | **60** | **120** |
| **O_2_ plasma** | 7.7 × 10^8^ ± 1.2 × 10^8^ | 1.9 × 10^6^ ± 1.8 × 10^6^ |  | 3.1 × 10^5^ ± 2.4 × 10^4^ | 5.9 × 10^4^ ± 8.5 × 10^4^ | 1.5 × 10^4^ ± 1.9 × 10^4^ |
| **O_2_ plasma/UV** | 7.7 × 10^8^ ± 1.2 × 10^8^ | 1.3 × 10^6^ ± 1.6 × 10^6^ |  | 1.5 × 10^5^ ± 2.3 × 10^5^ | 1.5 × 10^4^ ± 1.4 × 10^4^ | 3.3 × 10^2^ ± 5.2 × 10^2^ |
| **O_2_ plasma/UV (vapor)** | 7.7 × 10^8^ ± 1.2 × 10^8^ | 3.5 × 10^5^ ± 4.3 × 10^5^ |  | 7.3 × 10^4^ ± 1.6 × 10^5^ | 1.6 × 10^4^ ± 1.7 × 10^4^ | 0 |
| **Persister cells** |  |  |  |  |  |  |
| **Time (sec)** | **0** | **20** | **30** | **40** | **60** | **120** |
| **O_2_ plasma** | 5.6 × 10^8^ ± 3.3 × 10^8^ | 1.0 × 10^4^ ± 1.2 × 10^4^ | 2.4 ×10^4^ ± 2.8 × 10^4^ | 4.2 × 10^4^ ± 5.8 × 10^4^ | 1.1 × 10^4^ ± 1.6 × 10^4^ | 2.0 × 10^3^ ± 3.8 × 10^3^ |
| **O_2_ plasma/UV** | 6.0 × 10^8^ ± 3.2 × 10^8^ | 3.1 × 10^4^ ± 3.7 × 10^4^ | 1.1 × 10^4^ ± 1.8 × 10^4^ | 1.7 × 10^4^ ± 2.9 × 10^4^ | 5.4 × 10^3^ ± 6.9 × 10^3^ | 2.1 × 10^1^ ± 4.9 × 10^1^ |
| **O_2_ plasma/UV (vapor)** | 6.0 × 10^8^ ± 3.2 × 10^8^ | 4.7 × 10^4^ ± 5.6 × 10^4^ | 2.1 × 10^4^ ± 2.8 × 10^4^ | 2.3 × 10^4^ ± 2.2 × 10^4^ | 1.2 × 10^4^ ± 1.7 × 10^4^ | 4.4 × 10^2^ ± 7.9 × 10^2^ |

**Supplemental Table 4. *Escherichia coli* biofilm removal by RVR.** The well number of growths after treatment in UV, UV cover, O_2_ plasma, O_2_ plasma/UV, and O_2_ plasma/UV without cover mode. Non-treatment (NT) is indicated as a control. The graph of these data is shown in **Fig. 5**.

|  | **NT** | **UV** | **UV cover** | **O_2_ plasma** | **O_2_ plasma/UV** | **O_2_ plasma/UV w/o cover** |
| --- | --- | --- | --- | --- | --- | --- |
| **Well number of growths** | 96 ± 0 | 2 ± 2 | 19 ± 14 | 53 ± 24 | 16 ± 14 | 0 ± 0 |


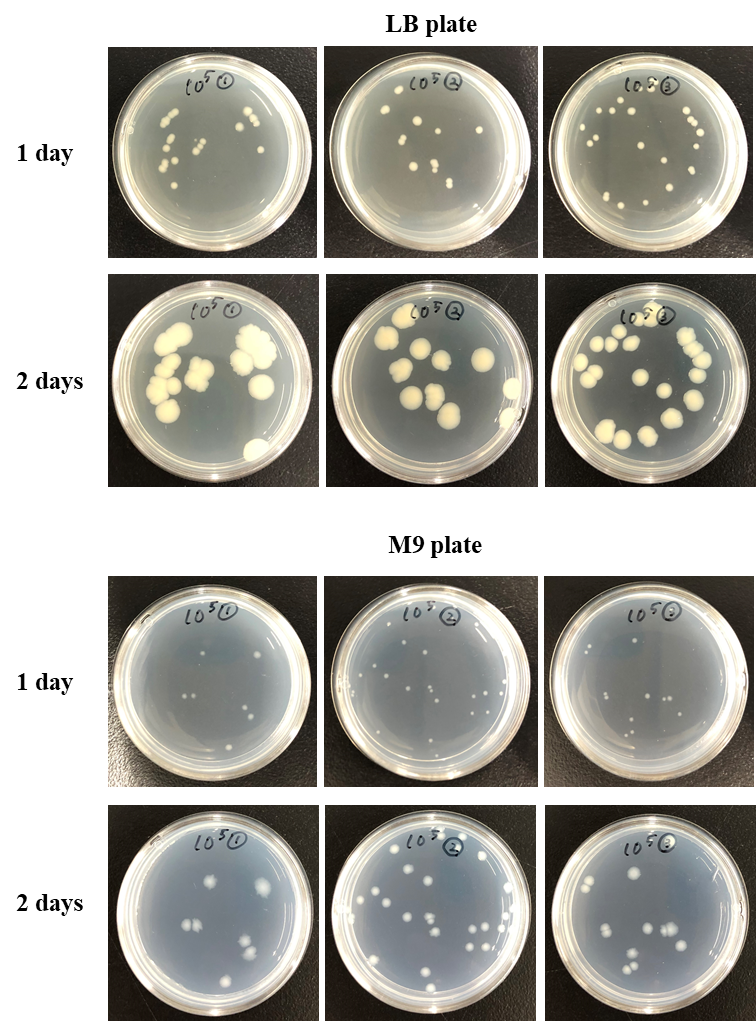


**Supplemental Figure 1**

**
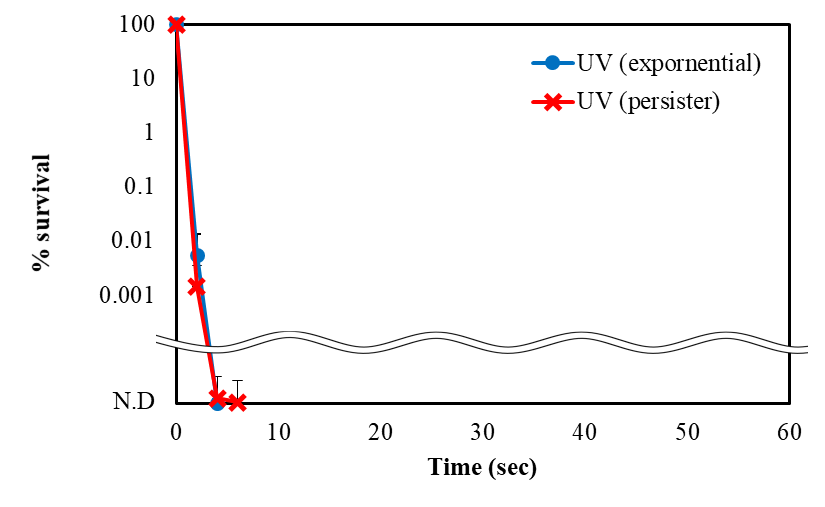
**

**Supplemental Figure 2**

**
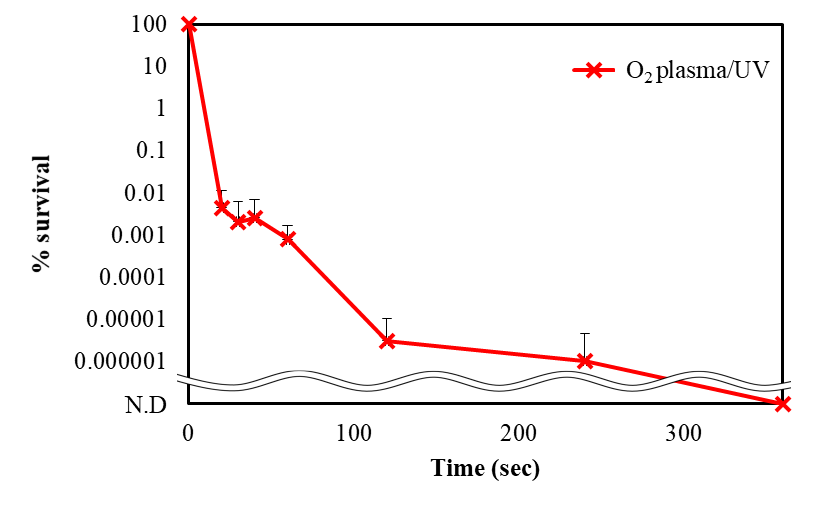
**

**Supplemental Figure 3**
